# Epithelioid sarcoma arising from the temporal space: A case report

**DOI:** 10.1097/MD.0000000000012529

**Published:** 2018-09-21

**Authors:** Yong Seek Kim, Hyo Sung Kwak, Gyung Ho Chung, Yo Na Kim, Seungbae Hwang

**Affiliations:** a Department of Radiology, Chonbuk National University Medical School and Hospital; b Research Institute of Clinical Medicine of Chonbuk National University-Biomedical Research Institute of Chonbuk National University Hospital; c Department of Pathology, Chonbuk National University Medical School and Hospital, Jeonju, Republic of Korea.

**Keywords:** epithelioid sarcoma, head, neoplasms, soft tissue

## Abstract

**Introduction:**

Epithelioid sarcoma is a malignant soft tissue tumor arising from mesenchymal tissue and usually occurs in the extremities. The tumor involving the head and neck region is extremely rare. We present radiologically well-documented case of an epithelioid sarcoma arising from the temporal space.

**Case presentation:**

A 35-year-old woman presented with a slowly growing, painless palpable mass in the left temporal area. Ultrasound (US) revealed a lobulated hypoechoic mass with internal vascularity. On magnetic resonance (MR) imaging, the mass showed heterogeneous signal intensity with a central necrotic area and peritumoral infiltration. On the basis of the clinical and radiological characteristics, the lesion was considered to be a malignant tumor originating from soft tissue. An incisional biopsy was performed. The diagnosis of epithelioid sarcoma was based on microscopic examination and immunohistochemical analysis. ^18^F-fluorodeoxyglucose positron emission tomography/computed tomography (^18^FDG-PET/CT) was used to stage the tumor and demonstrated intense FDG uptake in the mass without regional lymph node or distant metastasis. After the pathologic diagnosis of epithelioid sarcoma, the patient underwent total surgical resection of the tumor followed by postoperative irradiation. There was no evidence of recurrent disease during the follow-up period of 18 months.

**Conclusion:**

An epithelioid sarcoma should be considered in the differential diagnosis of a locally aggressive lesion occurring in the temporal space of head and exhibiting a heterogeneous appearance on imaging studies, including a central necrotic area and signal intensity suggestive of infiltration of soft tissue adjacent to the tumor. It is, however, true that head-and-neck involvement is very rare, and the radiological findings are not pathognomonic.

## 1 Introduction

Epithelioid sarcoma is a rare mesenchymal tumor accounting for 1% of all soft tissue sarcomas. Epithelioid sarcoma was first described and established as a distinct clinicopathological entity by Enzinger in 1970.^[1]^ The tumor is an aggressive, malignant soft tissue tumor that typically occurs in the distal portions of the extremities of young adults; involvement of the head-and-neck region is extremely rare.^[2]^ The tumor principally presents as a subcutaneous or dermal mass that spreads along the fasciae and tendon sheaths.^[1,3]^ Although several reports on tumor features evident on computed tomography (CT) and/or magnetic resonance (MR) imaging have appeared, the radiological findings of epithelioid sarcoma are variable. No characteristic features allowing the tumor to be differentiated from other malignant soft tissue tumors have been defined to date. The present study reports a case of epithelioid sarcoma presenting as a subcutaneous mass in the temporal space. We also briefly review the literature. To our knowledge, no tumor in this location has been imaged to the extent that we report.

## 2 Case report

**Figure 1:**
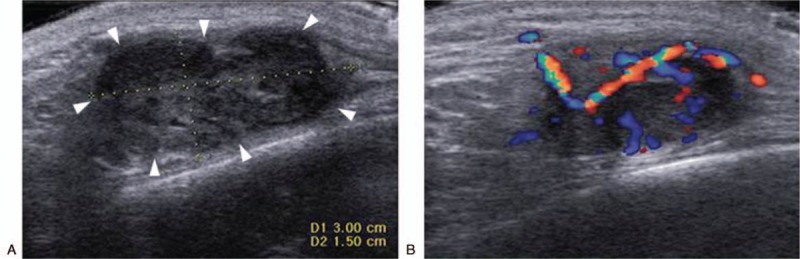
Gray-scale (A) and color Doppler (B) ultrasonographic images show a somewhat ill-demarcated, lobulated hypoechoic mass (arrowheads) located subcutaneously in the left temporal region; the extent of internal vascularity is moderate.

**Figure 2:**
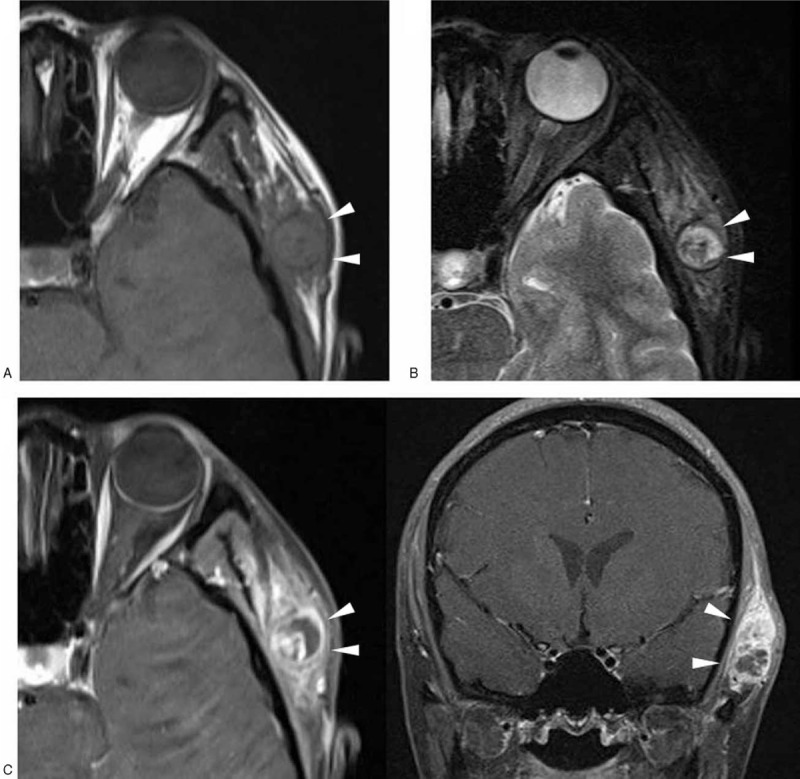
Magnetic resonance images reveal an ill-demarcated soft tissue mass (arrowheads) located in the temporal scalp between the temporal fascia and the temporalis muscle, exhibiting a homogeneous hypo- to isointense signal on a T1-weighted image (A); a heterogeneous mixture of iso- and hyperintense signals on a T2-weighted image (B); and heterogeneous intense enhancement with peritumoral infiltration-like signal changes along the adjacent soft tissues (including the temporal fascia and the superficial portion of the temporalis muscle) on fat-suppressed T1-weighted images taken after intravenous administration of gadolinium (C).

A 35-year-old woman presented with a 1-year history of a progressively growing painless mass in the left temporal area. Physical examination revealed a firm, nontender palpable mass in the left temporal area just superior to the zygomatic arch. The lesion exhibited no superficial ulceration or cutaneous erosion. All laboratory data were normal. Initial work-up with ultrasound (US) (Accuvix XG; Medison, Seoul, Korea) revealed a somewhat ill-demarcated, lobulated hypoechoic mass 3.0 × 2.5 × 1.5 cm in dimensions located subcutaneously in the left temporal region (Fig. 1A). Color Doppler US revealed moderate internal vascularity, without pulsatility (Fig. 1B). To further evaluate the lesion, the patient was examined using a 3.0-Tesla MR scanner (Verio; Siemens, Erlangen, Germany) 10 days later. The mass yielded a hypointense-to-isointense signal relative to that of the adjacent muscles on T1-weighted imaging (Fig. 2A), and a heterogeneous mixture of isointense and hyperintense signals (with a central dark spot) on T2-weighted imaging (Fig. 2B). After intravenous administration of gadolinium contrast material, the mass exhibited heterogeneous intense enhancement with the nonenhancing portion indicating internal necrosis (Fig. 2C). The mass was located in the left temporal scalp, between the temporal fascia and the temporalis muscle, and had a multilobulated margin. T2-weighted and contrast-enhanced imaging revealed diffuse, high-level signaling abnormalities extending from the mass into the surrounding soft tissue, spreading along the temporal fascia and the temporalis muscle. However, there was no evidence of bone or bone marrow involvement.

**Figure 3:**
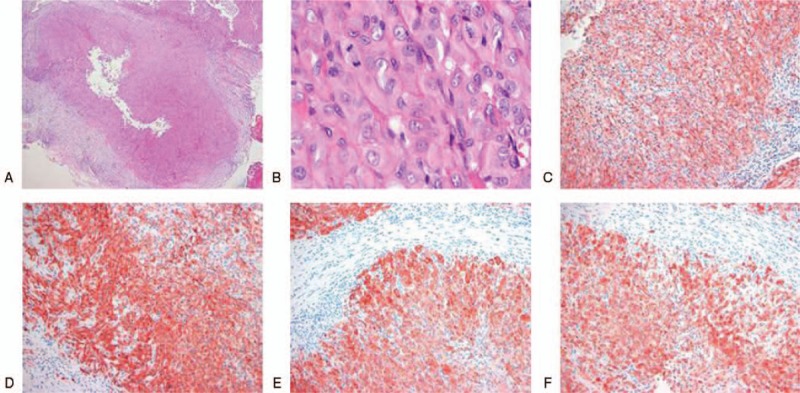
Histopathological examination reveals (A) tumor cells aggregating in nodules exhibiting central necrosis, affording a pseudogranulomatous appearance (hematoxylin and eosin stain, ×40). (B) The tumor cells are epithelioid in nature with abundant eosinophilic cytoplasm, vesicular chromatin, and small nucleoli (hematoxylin and eosin stain, ×400). Immunohistochemical staining shows that the cells are positive for both mesenchymal markers [smooth muscle actin (C) and CD34 (D)] and epithelial markers [cytokeratin (E) and epithelial membrane antigen (F)] (original magnification, ×200).

On the basis of the clinical and radiological characteristics, the lesion was considered to be a malignant tumor originating from soft tissue. An incisional biopsy was performed. Histologically, the tumor cells were aggregated in nodules exhibiting central necrosis, thus appearing pseudogranulomatous in nature (Fig. 3A). The tumor cells were epithelioid, with abundant eosinophilic cytoplasm, vesicular chromatin, and small nucleoli (Fig. 3B). Immunohistochemical analysis showed that the tumor cells stained positive for both mesenchymal markers [smooth muscle actin (Fig. 3C) and cluster of differentiation (CD) 34 glycoprotein (Fig. 3D)] and epithelial markers [cytokeratin (CK) (Fig. 3E) and epithelial membrane antigen (EMA) (Fig. 3F)]. The tumor cells were not immunoreactive for CD 31, S-100 protein, Melan-A, MyoD1, desmin, or CD 68. The lesion was pathologically diagnosed as an epithelioid sarcoma.

**Figure 4:**
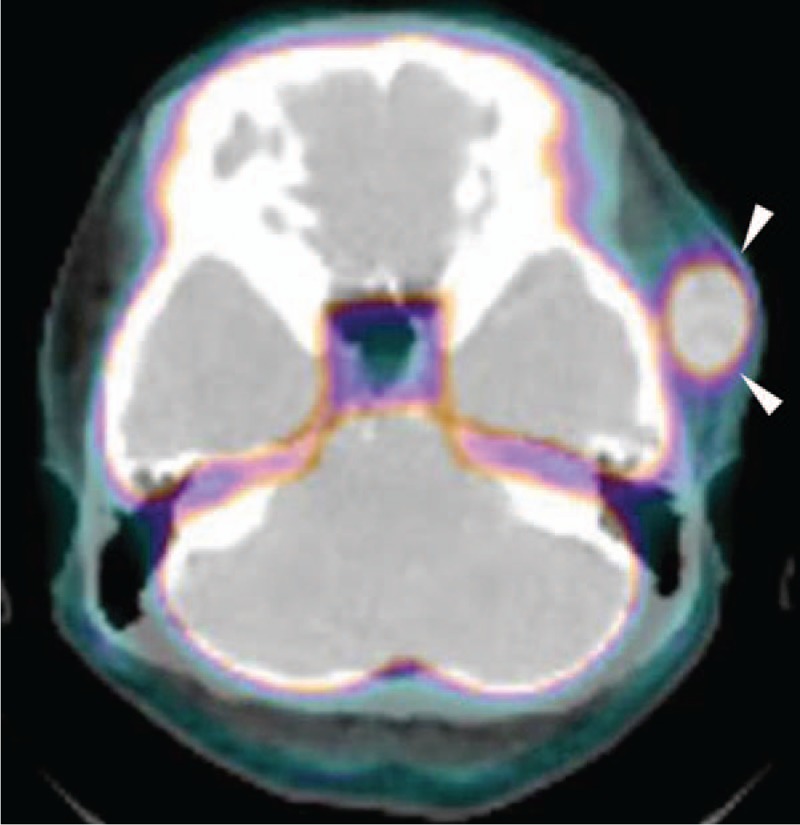
^18^F-fluorodeoxyglucose positron emission tomography/computed tomography shows an FDG-avid soft tissue lesion (arrowheads) in the left temporal space (maximum SUV = 10.78).

^18^F-fluorodeoxyglucose positron emission tomography/CT (^18^FDG-PET/CT) (Biograph Truepoint 40; Siemens, Berlin, Germany) was used to stage the tumor and revealed focally intense FDG uptake (Fig. 4) but no regional lymph node or distant metastasis. The patient underwent total surgical resection of the tumor followed by postoperative irradiation. In cross-section, the tumor was a pale gray solid mass containing hemorrhagic and necrotic portions. There was no evidence of recurrent disease during the follow-up period of 18 months. The present study was approved by the institutional review board of Chonbuk National University Hospital and written informed consent for the publication of the case was obtained from the patient.

## 3 Discussion

An epithelioid sarcoma is an aggressive soft tissue sarcoma characterized by variation in all of clinical presentation, imaging findings, and histological features.^[4]^ Histologically, classical epithelioid sarcoma features nodular proliferation of polygonal or spindle-shaped epithelioid cells with eosinophilic cytoplasm and pleomorphic nuclei, and areas of central degeneration and necrosis.^[5,6]^ Such sarcomas occur predominantly in distal parts of the extremities with a predilection for the hands and forearms.^[5]^ In 1997, a proximal type of epithelioid sarcoma was identified by Guillou et al^[7]^; this occurs principally in the chest wall, thigh, pelvis, and genital tract,^[7,8]^ and is histologically characterized by the presence of large or rhabdoid cells with vesicular nuclei and prominent nucleoli.^[5,7]^ The head-and-neck regions are rarely affected, accounting for 1% of all cases.^[2]^ Such tumors occur in the scalp, the auricle and periauricular area, the neck, the temporo-mandibular region, the oral cavity, the parotid gland, the nose, and the orbit.^[6,9–13]^ Epithelioid sarcoma is often characterized by a high propensity toward regional lymph node metastasis, local recurrence, and distant metastasis. The prognosis in terms of overall survival is associated with all of tumor location; tumor size and depth; the number of mitotic figures; and the presence/absence of hemorrhage, necrosis, and vascular invasion.^[2,4,5]^

Several reports on the imaging characteristics of epithelioid sarcoma have appeared. Most radiological findings are MR images and are found in various case reports. Only a few studies on MR imaging findings in a series of patients with epithelioid sarcoma have appeared; no obvious characteristic MR features have been documented.^[4,8]^ The studies show that variations in tumor appearance on MR images are attributable to the diverse histological morphologies of the tumors. Tateishi et al^[8]^ reported on the radiological manifestations of 16 patients with proximal-type tumors, and suggested that it may be difficult to differentiate the 2 types of epithelioid sarcoma using MR imaging features only. The cited authors observed that most lesions (87.5%) were multinodular in appearance, or exhibited multilobulated contours. Such patterns were suggestive of epithelioid sarcoma, but it was crucial to distinguish such sarcomas from other soft tissue sarcomas, although smaller lesions were round and well-circumscribed. Histologically, the tumors exhibited varying degrees of cellularity, necrosis, fibrosis, and hyalinization.^[4]^ In particular, central degeneration or necrosis was commonly apparent^[2,7]^; this contributed significantly to the tumor enhancement pattern evident upon postcontrast imaging. In our case, the tumor was clearly multilobulated in nature and exhibited nonenhancing portions on postcontrast images, corresponding to the hyperintense areas apparent on T2-weighted images, and to pathologically proven regions of intratumoral necrosis.

In gross section, epithelioid sarcoma usually exhibits indistinct infiltrating margins.^[2]^ In the context of treatment, it is critical to distinguish peritumoral infiltration or invasion from reactive inflammatory change when soft tissue adjacent to the tumor exhibits signaling abnormalities upon MR imaging. In the present case, a heterogeneous increase in the signal intensity of soft tissue adjacent to the tumor was evident on both T2-weighted and postcontrast images. Pathologically, this corresponded to regions of tumor infiltration. This finding is in contrast to what was noted by Hanna et al^[4]^, who found that a peritumoral, edema-like hyperintense signal on T2-weighted images corresponded, histologically, to an inflammatory reaction.

On the basis of previous imaging studies, regional lymph node metastasis is common (50–62.5% of cases),^[4,8]^ but calcification, hemorrhage, or bone involvement is infrequent.^[4,8,14]^ Our present case did not exhibit nodal involvement, calcification, or bone invasion either radiologically or pathologically. However, hemorrhagic tumor components were evident in the specimen cross-section. T2-weighted imaging showed regions of low signal intensity within the tumor; these may correspond to the hemorrhagic foci that were pathologically documented.

Although the radiological findings thus do not permit a specific diagnosis of epithelioid sarcoma, immunohistochemical analysis is useful in this context. Epithelioid sarcomas are commonly positive for CK, vimentin, and EMA, and for CD 34 in about half of all cases.^[2,6]^ However, most tumors are negative for S-100 protein.^[6]^ In the present study, the tumor was positive for CK, vimentin, EMA, and CD 34, but negative for S-100 protein, consistent with the data of previous studies.

In conclusion, we present here an epithelioid sarcoma arising from the temporal space. An epithelioid sarcoma should be considered in the differential diagnosis of a locally aggressive lesion that is heterogeneous in appearance on imaging, although the head-and-neck region is rarely involved and radiological findings are not pathognomonic. A multilobulated tumor contour and a central necrotic area may aid in distinguishing an epithelioid sarcoma from other soft tissue sarcomas. In addition, an abnormal signal from soft tissue adjacent to the tumor suggests peritumoral infiltration.

## Author contributions

**Conceptualization:** Seungbae Hwang.

**Data curation:** Yong Seek Kim, Yo Na Kim.

**Formal analysis:** Seungbae Hwang.

**Investigation:** Yong Seek Kim.

**Resources:** Yo Na Kim.

**Supervision:** Hyo Sung Kwak, Gyung Ho Chung, Seungbae Hwang.

**Validation:** Hyo Sung Kwak, Gyung Ho Chung.

**Writing – original draft:** Yong Seek Kim.

**Writing – review & editing:** Hyo Sung Kwak, Gyung Ho Chung, Seungbae Hwang.

Seungbae Hwang orcid: 0000-0002-2420-3182
